# Limits of temperature adaptation and thermopreferendum

**DOI:** 10.1186/s13578-021-00574-9

**Published:** 2021-04-06

**Authors:** K. B. Aslanidi, D. P. Kharakoz

**Affiliations:** Institute of Theoretical and Experimental Biophysics, Russian Academy of Sciences, Pushchino, Moscow region Russia 142290

**Keywords:** Adaptation, Temperature, Cold shock, Heat shock, Thermopreferendum, Viability polygon, Lipids

## Abstract

**Background:**

Managing the limits of temperature adaptation is relevant both in medicine and in biotechnology. There are numerous scattered publications on the identification of the temperature limits of existence for various organisms and using different methods. Dmitry Petrovich Kharakoz gave a general explanation for many of these experimental results. The hypothesis implied that each cycle of synaptic exocytosis includes reversible phase transitions of lipids of the presynaptic membrane due to the entry and subsequent removal of calcium ions from the synaptic terminal. The correspondence of the times of phase transitions has previously been experimentally shown on isolated lipids in vitro. In order to test the hypothesis of D.P. Kharakoz in vivo, we investigated the influence of the temperature of long-term acclimatization on the temperature of heat and cold shock, as well as on the kinetics of temperature adaptation in zebrafish. Testing the hypothesis included a comparison of our experimental results with the results of other authors obtained on various models from invertebrates to humans.

**Results:**

The viability polygon for Danio rerio was determined by the minimum temperature of cold shock (about 6 °C), maximum temperature of heat shock (about 43 °C), and thermopreferendum temperature (about 27 °C). The ratio of the temperature range of cold shock to the temperature range of heat shock was about 1.3. These parameters obtained for *Danio rerio* describe with good accuracy those for the planarian *Girardia tigrina*, the ground squirrel *Sermophilus undulatus*, and for* Homo sapiens*.

**Conclusions:**

The experimental values of the temperatures of cold shock and heat shock and the temperature of the thermal preferendum correspond to the temperatures of phase transitions of the lipid-protein composition of the synaptic membrane between the liquid and solid states. The viability range for zebrafish coincides with the temperature range, over which enzymes function effectively and also coincides with the viability polygons for the vast majority of organisms. The boundaries of the viability polygon are characteristic biological constants. The viability polygon of a particular organism is determined not only by the genome, but also by the physicochemical properties of lipids that make up the membrane structures of synaptic endings. The limits of temperature adaptation of any biological species are determined by the temperature range of the functioning of its nervous system.

## Introduction

Any living organism can exist only within a limited range of the ambient temperatures. The limits of the temperature adaptation are of particular importance for ectotherms – organisms that cannot control their body temperature and constantly have to adapt to the changes of the ambient temperature. In the course of adaptation, the lipid composition and physicochemical properties of membranes in ectotherms undergo considerable changes [1, 2]. During this adaptation process, neurons support unique lipid compositions with specific physicochemical properties [2]. Moreover, the composition and physicochemical properties of membrane lipids in ectotherms depend on the ambient temperature [1]. From early work on model membranes, it was known that the temperature of the phase transition of lipid membranes from the state of a liquid crystal to a solid gel state depends on the concentration of Ca^2+^ions [3, 4].

Dmitry Petrovich Kharakoz was the first to draw attention to the fact that each cycle of synaptic exocytosis includes reversible phase transitions of lipids of the presynaptic membrane due to the entry and subsequent removal of calcium ions from the synaptic terminal [5, 6]. According to this notion, lipid membrane has to be in a liquid–crystal state close to the phase transition temperature, and adaptation to various temperatures has to occur through the appropriate alterations in the lipid composition of the presynaptic membrane and synaptic vesicles. The hypothesis implied that the Ca^2+^-induced chain-ordering phase transition in the lipid component of synaptic membranes plays a key role in the neurotransmitter release [5]. It was proposed that the plasma membrane in the active zones of synaptic terminals contains self-assembling cooperative domains whose Ca^2+^-induced solidification may be the driving force of the fast neurotransmitter release in the central synapses [6]. It was shown that the phase-transitional mechanism can provide a high rate of synaptic exocytosis known for the fastest synapses in the central nervous system [7]. The important role of the mechanisms of fusion and breaking of synaptic membranes for the functioning of neural networks was noted by other authors [8, 9], however, they did not pay due attention to phase transitions in membranes. It should be noted that both approaches describe the disturbances in the action potential propagation at the level of presynaptic [5, 6] or postsynaptic [10] membranes and ascribe a key role to the alterations in the phase state of the lipid-protein compositions of the neuronal membrane [11–13]. Considering the temperature dependence of the intracellular Ca^2+^ content revealed earlier on the simplest models of cell's ionic-osmotic homeostasis [14, 15], the existence of the upper and lower temperature limits for synaptic exocytosis can be expected. At a temperature higher than the upper limit the presynaptic membrane will remain in a liquid state even during the action potential causing an increase in the intracellular Ca^2+^ concentration. Cooling the membrane to the temperatures lower than the lowest limit will also upset the synaptic conduction, as at any cytoplasmic concentration of Ca^2+^ the membrane will remain in a solid state. In both cases the neurotransmitter release will be stopped, and at the level of the whole organism this disturbance in the synaptic transmission can be recorded as a loss of righting reflex. The relation between the fluidity of the synaptic membranes and the loss of righting reflex was shown long ago [16]. Loss of righting reflex observed in the presence of narcotic drugs soluble in membrane lipids was explained by an increase in the membrane fluidity leading to disturbances of the nerve excitation conduction [16–18]. The mechanisms of the reversible assembly of synaptic vesicles at physiological temperature are generally understood [19]; however, temperature dependencies of the synaptic exocytosis are virtually unexplored. It is obvious that such investigations should be conducted on ectothermic animals that are able to survive in a wide range of temperatures. On the other hand, as far as no specific genes or neuroendocrine factors for hypometabolic states have been discovered by far, it is conceivable that endotherms, including humans, have a potential for this physiological state as well. Hypometabolism in newborn mammalians can serve a model for creation of such states in adult mammalians, including humans, for medical applications and for space exploration programs [20].

In order to test the hypothesis of D.P. Kharakoz in vivo, we investigated the influence of the temperature of long-term acclimation on the temperature of heat and cold shock, as well as the kinetics of temperature adaptation in zebrafish. Zebrafish *Danio rerio* is a typical exothermic animal that lives in a wide range of temperatures. The aim (goal) of this work was to identify the temperature range in which *Danio rerio* could survive and to compare these parameters with the results obtained in other biological species.

The experiment identified the temperatures at which the fish retains mobility driven by coordinated functions of the nerve and muscular systems. It is known that extreme changes in environmental temperatures cause a set of adaptive reactions that deteriorate the homeostasis of the body and lead to cold or heat shock. The stress temperature in exotherms depends on the adaptation temperature. For this purpose, we analyzed the dependence of the temperature, at which the synaptic transmission was disrupted (shock temperature), on ambient temperatures and adaptation time. The functional state of the synaptic transmission was assessed using the methodology of the loss of righting reflex [16]. It was assumed that at a shock temperature, intracellular changes in the content of calcium ions cannot cause a phase transition of neuronal membranes from a liquid crystalline state to a gel or vice versa [21, 22]. Testing the hypothesis included a comparison of our experimental results with the results of other authors obtained on various models from invertebrates to humans.

## Experimental model and methods

### Experimental model

Zebrafish (*Danio rerio)* was used as a typical ectothermic animal. An advantage of these fishes is an enormous amount of scientific literature and a completely deciphered genome. The nervous system of zebrafish is well characterized and their genome exhibits a high degree of homology with the genome of mammalians; therefore, it is widely admitted that these fishes can serve a useful model for medical and biological investigations [23–26], and in particular, for experimental studies of genetic and physiological mechanisms of stress, depressive behavior [21, 22], and other psychotic pathologies [27–29]. More than 1200 lines of *Danio rerio* are now available in appropriate genetic centers, as well as more than 10,000 specimens of frozen sperm, including that from more than 200 mutant and transgenic lines [24, 25]. An important advantage of these fishes is their small size, which makes it possible to neglect the temperature gradient across the body width in the experimental studies of the synaptic transmission blockade after a sharp change of the ambient temperature [30]. At the whole organism level the impairment of the synaptic transmission is recorded as a loss of righting reflex. The upper and lower limits for the functioning of the synaptic exocytosis are determined as a heat shock temperature T_H_ and a cold shock temperature T_C_, respectively. All parameters of the experiment were carefully maintained as the susceptibility to stress in zebrafish depends on environmental changes [31].

### Loss of righting reflex metodology

To study the dependence of T_H_ and T_C_ on the temperature of a prolonged acclimation T_A_, fishes *Danio rerio* (size 20–30 mm) were acclimated at various temperatures ranging from 9 to 38 °C for 7–10 days. The daylight duration was 12 h. Then the fishes were placed into an actively aerated transparent cylindrical 600 mL vessel with a diameter of about 10 cm. Air bubbles created a vertical flow of water along the axis of the cylinder. Water temperature was set significantly higher or lower than the temperature of the prolonged acclimation. The aeration intensity was adjusted so that in the normal conditions (that is, at any temperature not inducing loss of righting reflex) the fishes could maintain stability in the water flow created by the air bubbles. The temperature, at which the loss of righting reflex was observed within one minute after a sharp change of the ambient temperature, was defined as T_H_ or T_C_ [30]. The recording time (1 min) was chosen on the basis of the fact that within one minute the temperature gradients between water in the test chamber and the fish internal organs disappeared, while the cells' ionic composition and, the more so, the membrane lipid composition remained unchanged. Indeed, in our experiments, at a maximal temperature difference between the test and acclimation chambers of about 10 °C and a fish body width of 1.5–2.0 mm, the temperature gradients disappeared within tens of seconds. Regardless of the testing results, after the lapse of 1 min the fish was relocated back from the test chamber to the acclimation chamber with T_A_, the righting reflex recovered within seconds. If the fish retained the mobility in the water flow within 1 min, the temperature in the test chamber was increased by 1 °C, if the heat shock (T_H_) was tested, or lowered by 1 °C, if the cold shock (T_C_) was tested. In each experiment, 20 to 50 fishes were used.

In addition, the kinetics of adaptation of fishes to different temperatures was studied. The adaptation kinetics was evaluated by changes in T_H_ and T_C_ after a sharp change of T_A_. For this purpose, after a prolonged acclimation at fixed temperatures for 5–10 days, fishes were transferred to another acclimation chamber with different temperature. All chambers were aerated. The dependencies of temperatures T_H_ and T_C_ on the time of adaptation to a new temperature was recorded in the range from 5 min to 5–7 days. Each fish was used in the experiment no more than once a day.

The values of T_H_ and T_C_ and the measurement errors were determined using the Microsoft Office Excel 2007 software on the basis of a mean value and half width of the obtained distribution of the experimental temperatures.

### Registration of the thermopreferendum temperature

If the limits of temperature adaptation determine the range of temperatures in which the organism able to survive, then the temperature of the thermopreferendum is chosen by the organism independently in the presence of a wide choice of temperatures.

The thermopreferendum temperature was determined in a gradient bath 200 cm long and rectangular section 20 cm × 20 cm, divided into 8 compartments by transparent walls with a holes in the center of about 5 cm in diameter. All sections were aerated; in the first compartment the temperature was held at 18 °C and in the eighth compartment, at 32 °C. Fishes *Danio rerio* with size of 20–30 mm were placed into a gradient chamber after a 5-day acclimation at 18 °C or at 32 °C. Individual trajectories of fishes were obtained in separate experiments, in which the influence of the social behavior was excluded [32].

## Results

### Heat shock and cold shock

**Fig. 1:**
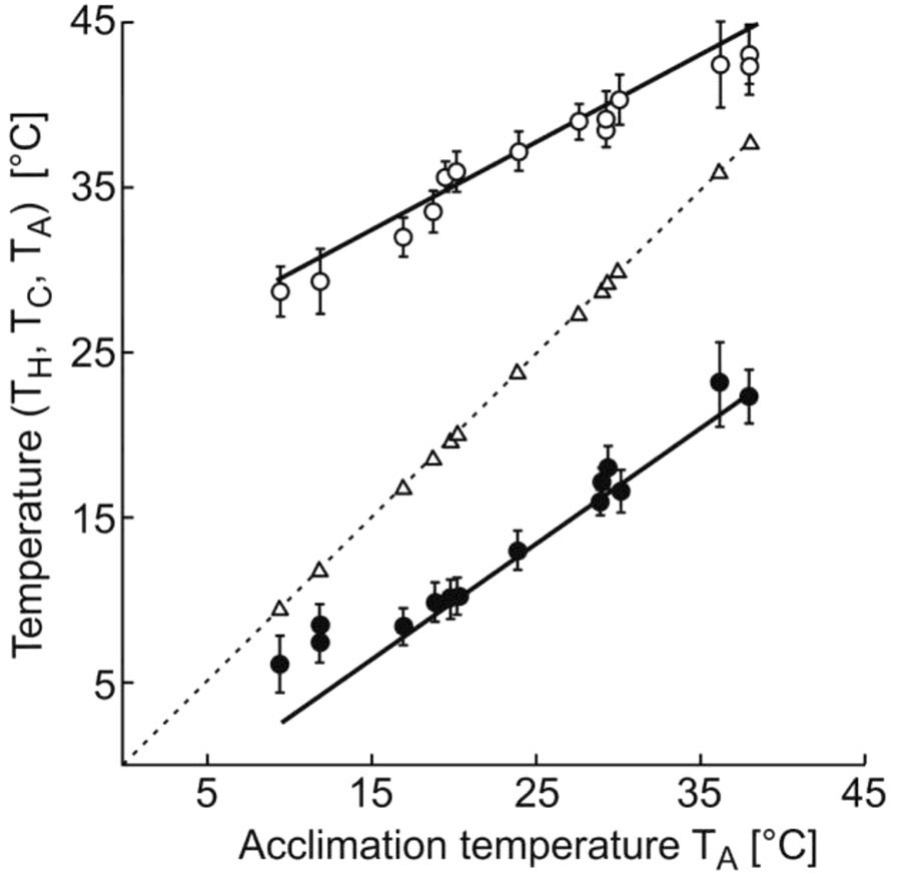
The dependencies of the heat shock temperature T_H_ and of the cold shock temperature T_C_ on the temperature of the long-term acclimation T_A_ in *Danio rerio*. Experimental values of T_C_ are shown by filled circles (●); values of T_H_, by *open circles* (○), and values of T_A_, by *open triangles* (Δ). Linear regression lines are drawn through temperature values in the range from 20 to 30 °C

The dependencies of the heat shock temperature T_H_ and of the cold shock temperature T_C_ on the temperature of the long-term acclimation T_A_ are presented in Fig. 1.

The difference between the cold shock temperature T_**C**_ and the acclimation temperature T_A_ varied from 5 °C to 12 °C, and the difference (T_H_−T_A_) varied from 5 °C to 20 °C. Upon return to the vessel with the temperature equal to T_A_ the righting reflex recovered within seconds. In the range of definitely tolerable acclimation temperatures, between 20 and 30 °C, both heat-shock temperatures T_H_ and cold-shock temperatures T_C_ linearly grew with an increase of the acclimation temperature T_A_: T_C_ = 0.70 T_A_ − 4.12, R^2^ = 0.98, T_H_ = 0.53 T_A_ + 24.20, R^2^ = 0.89.

The straight line T_A_ = T_A_ clearly demonstrates that the difference T_A_−T_C_ increases with increasing T_A_, and the difference T_H_−T_A_ decreases. However, the applied approach does not allow to determine the limit values T_C_ = T_A_ and T_H_ = T_A_. To identify both the minimum and maximum values of the shock temperatures, the registration of the kinetics of temperature adaptation was used.

### Kinetics of temperature adaptation

**Fig. 2:**
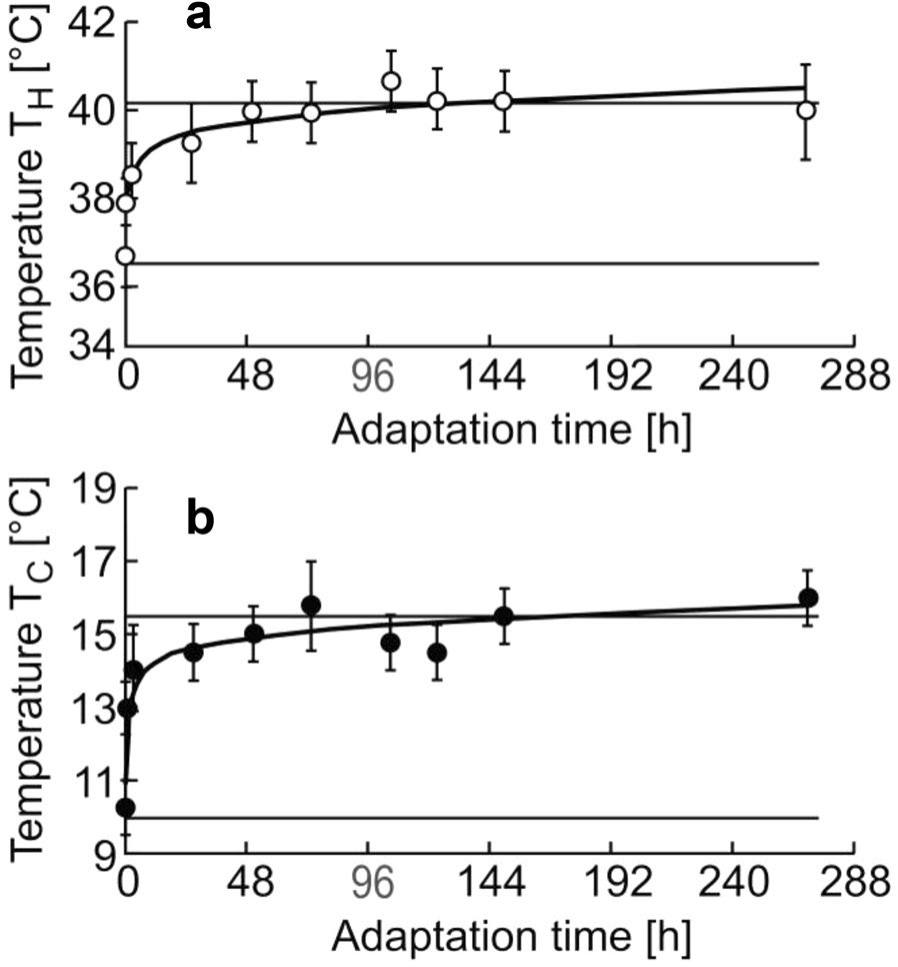
The adaptation kinetics of T_H_ and T_C_ upon sharp change of the water temperature from 20 to 30 °C of *Danio rerio*. Abscissa, the adaptation time, hours. Experimental values of the heat shock temperature, T_H_, are shown by *open circles* (○) and the values of cold shock, T_C_, by *filled circles* (●). Horizontal lines indicate the values of T_H_ and T_C_ after a prolonged acclimation at 20 °C and at 30 °C

The adaptation kinetics of T_H_ and T_**C**_ upon sharp change of the water temperature from 20 °C to 30 °C are presented in Fig. 2.

**Fig. 3:**
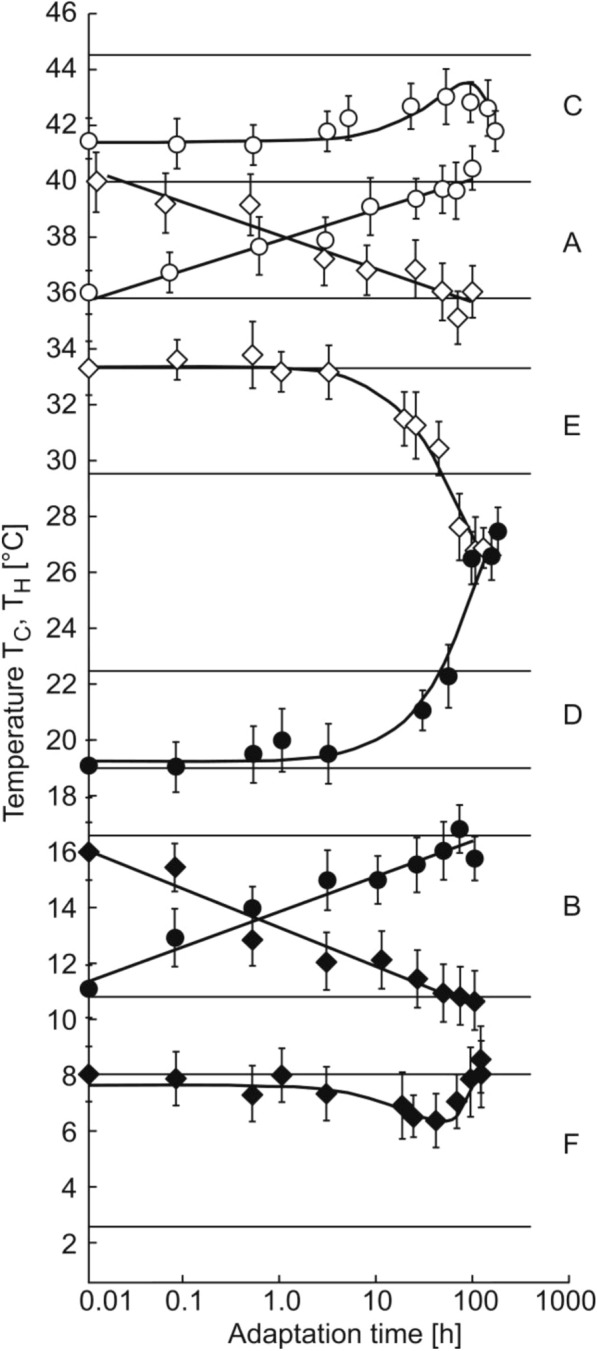
The kinetics of temperature adaptation of *Danio rerio. Abscissa*, adaptation time (t) in the logarithmic scale; ordinate, shock temperatures T_**H**_(○), T_H_(◊), T_C_(●), T_C_(♦). Experimental values of T_H_ are shown by open symbols (○ and ◊) and values of T_C_, by filled (black) symbols (● and ♦). The shock temperatures recorded after the transfer of the fishes into water with higher temperatures are designated with circles (○ and ●) and shock temperatures recorded after the transfer of the fishes into water with lower temperatures, with diamonds (◊ and ♦). *Horizontal lines* show the stationary values of T_H_ and T_C_ calculated on the basis of linear regression, with regard of the values of shock temperatures in the range from 20 °C to 30 °C: T_C_ = 0.70 T_A_—4.12, T_H_ = 0.53 T_A_ + 24.20. The letters A, B, C, D, E and F indicate the kinetics of adaptation in different temperature ranges

Both kinetics are characterized by a smooth transition of T_H_(○) and T_C_(●) to a new stationary level. The dependencies of T_H_(○) and T_C_(●) in the adaptation time in the logarithmic scale (lg(t)) were linear. The adaptation kinetics in the whole viability range of zebrafish is shown in Fig. 3. The experimental results presented in Fig. 3 indicate that upon a sharp change of the water temperature, new stationary values of T_H_ and T_C_ are established within several days.

Linear dependencies of T_H_(○) and T_H_(◊) on the logarithm of the adaptation time ln(t) upon the transfer of fishes in the range of definitely tolerable temperatures, from 20 °C to 30 °C and from 30 °C to 20 °C, are presented in Fig. 3a. Horizontal lines show the stationary values of the thermal shock T_H_ equal to 36 °C during prolonged acclimation at 20 °C and equal to 40 °C at 30 °C. The adaptation kinetics of T_C_(●) and T_C_(♦) upon the transfer of fishes from 20 °C to 30 °C and from 30 °C to 20 °C are presented in Fig. 3b. Horizontal lines indicate stationary values of the cold shock T_C_, equal to 11.0 °C and 16.5 °C for 20 °C and 30 °C, respectively. After the temperature shift from 20 °C to 30 °C the linear regressions took the form: T_H_ = 0.43 ln(t) + 38.01 at R^2^ = 0.96 and T_**C**_ = 0.52 ln(t) + 12.93 at R^2^ = 0.98. When the temperature was shifted back from 30 °C to 20 °C, the dependencies took the form: T_H_ = –0.48 ln(t) + 38.17, R^2^ = 0.91 and T_C_ = –0.57 ln(t) + 12.31, R^2^ = 0.95.

In the plots presented in Fig. 3a and b, changes in T_H_ and T_C_ within the first hour after a sharp change in the temperature amounted about a half of the whole range of the changes in the shock temperatures.

During adaptation to temperatures lower than 20 °C or higher than 30 °C, the dependencies of shock temperatures T_C_ and T_H_ on T_A_ departed from the linearity mentioned above. The kinetics of T_H_(○) recorded upon the transfer of fishes from 30 °C to 38 °C is presented in Fig. 3c. Horizontal lines show stationary values of heat shock temperatures of 40.0 °C and 44.5 °C, calculated on the basis of the equation T_H_ = 0.53 T_A_ + 24.2 for 30 °C and 38 °C, respectively. The kinetics was low-sloped, maximal value T_**H**_(○) = (T_H_)_max_ = 43 °C C was reached only on day 2–4. The kinetics of T_**C**_(●) upon the transfer of a fish from 30 °C to 38 °C is shown in Fig. 3d. Horizontal lines indicate calculated stationary values of cold shock temperatures of 19 °C and 22.5 °C for 30 °C and 38 °C, respectively. The T_C_(●) value remained unchanged for 2 days after transferring fishes to 38 °C, and then it began to grow rapidly; within one day the calculated level of 22.5 °C was reached, and three days later the maximum temperature of cold shock (T_C_)_max_, equal to 27 °C in zebrafish, was reached.

The kinetics of T_H_(◊) and T_C_(♦) upon the transfer of the fishes from 17 °C to 9.5 °C is presented in Fig. 3e and f. Horizontal lines indicate calculated stationary values of heat shock temperatures of 33 °C and 29.5 °C, as well as cold shock temperatures of 8 °C and 2.5 °C for 17 °C and 9.5 °C, respectively. The temperature T_C_(♦) practically did not change during the first day, and the minimum value of T_C_(♦) was reached only on days 2–4 and amounted to (T_C_)_min_ = 6.5 °C. During the adaptation to extremal temperatures, T_H_(◊) (Fig. 3e) and T_C_(●) (Fig. 3d) got beyond the expected level within one day and approached the temperature (T_H_)_min_ = (T_C_)_max_ = 27 °C. This temperature is of special importance for zebrafish.

### Thermopreferendum temperature

In our experiments in the gradient bath, fish *Danio rerio* chose the temperature 25–27 °C. The thermopreferendum temperature did not depend on the temperature of the prolonged acclimation either at T_A_ = 18 °C or T_A_ = 32 °C, and the decision-taking time did not exceed 1.0–1.5 h. All 10 fish remained at a chosen temperature and moved away for a few minutes not more than by 2–4 °C during the observation period of at least 4 h.

**Fig. 4:**
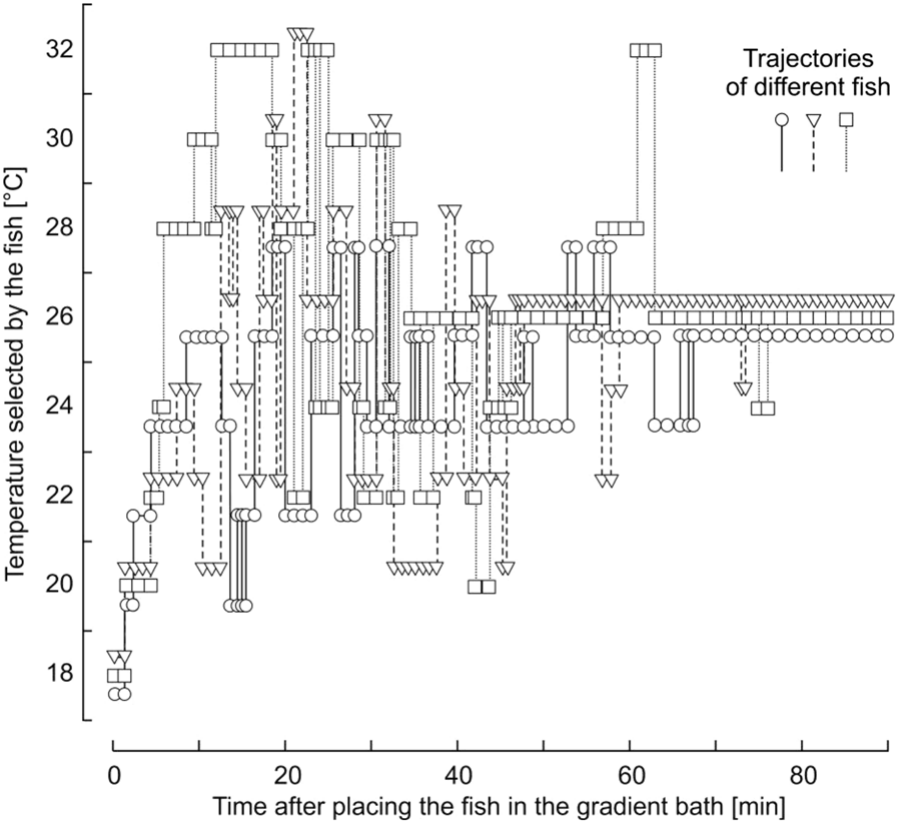
Temperature selection by three *Danio rerio* fish in a gradient bath. Abscisa, time after placing the fish in the gradient bath, min; ordinate, temperature selected by the fish, °C. Individual trajectories fish movements were obtained in separate experiments, excluding the influence of the flock, and are indicated by different symbols (□, Δ, Ο)

Figure 4, illustrating the temperature selection by three *Danio rerio* fish in a gradient bath recorded in separate experiments. Before the experiment, the fish were kept at 18 °C for 5 days.

At the boundaries of each section of the gradient bath, the water temperature differed from the temperature in the center of the section by 1 °C. The presence of a fish in the 26 °C section means that the fish avoids water with temperatures above 27 °C and below 25 °C. This means that the temperature of the thermopreferendum is in the range from 25 to 27 °C. All 10 fish chose the temperature of the thermopreferendum within 1 h and remained in this section for at least 4 h.

The thermopreferendum temperature T_PR_ for zebrafish in particular, is attractive due to the fact that at an acclimation temperature T_A_, equal to the T_TP_, the range of permissible warm temperatures equals to the range of permissible cold temperatures: T_PR_−T_C_ = T_H_ – T_PR_. This means that after a prolonged acclimation at T_A_ = 26 °C the fishes can equally easy overcome both cooling and heating by more than 10 °C: 26 °C—T_C_ ≈ T_H_ – 26 °C. At a temperature of T_A_ below 25 °C, the difference between T_H_ and T_A_ increased, and at a T_A_ above 27 °C, it decreased to zero at T_A_ = 43 °C. With T_A_ more than 27 °C, the difference between T_A_ and T_C_ increased, and with T_A_ less than 25 °C, the difference between T_A_ and T_C_ decreased to zero at T_A_ = 6.5 °C. This means that T_PR_ is the most comfortable and safest temperature for *Danio rerio*. These results are a scientifically based definition of a thermal preferendum.

### The viability polygon

**Fig. 5:**
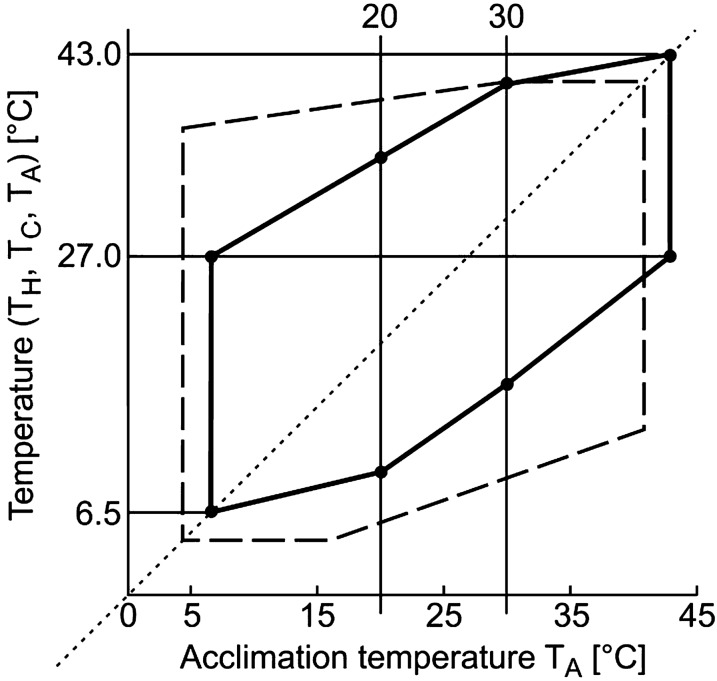
Comparison of the viability polygon obtained for *Danio rerio* by means of the loss of righting reflex methodology (*solid line*) and the polygon of tolerable temperatures obtained by the methodology of the critical maximal and critical minimal temperatures (*dashed line*) [34]. Abscissa, T_A_ [°C] and ordinate, T_H_ [°C], T_C_ [°C] and T_A_ [°C]

The viability polygon for *Danio rerio* presented in Fig. 5 is constructed on the basis of the linear dependencies of T_C_ and T_H_ on T_A_ in the T_A_ range from 20 to 30 °C. The values of T_H_ and T_C_ at 20 °C and 30 °C are connected by direct lines with the corresponding values obtained at extremal temperatures (T_C_)_min_ = 6.5 °C and (T_H_)_max_ = 43 °C.

It should be noted that the linear dependence of T_H_ on T_A_, obtained on the basis of the experimental results in the T_A_ range from 20 to 30 °C, adequately describes all the results for T_H_ in a wider range, from 7 to 30 °C. Similarly, a linear dependence of T_C_ on T_A_, obtained for a range from 20 to 30 °C adequately describes the results in the T_A_ range from 20 to 43 °C. Of interest is the fact that the range of tolerable temperatures from 20 to 30 °C lays halfway between the extremal values of T_A_: (20 °C – 7 °C) ≈ (43–30 °C).

## Discussion

### Heat shock and cold shock

In the in vivo behavioral experiments presented in our work, the impairment of coordination and mobility of the fish was recorded under extreme changes in body temperature equal to the ambient temperature. Previously, it was shown that the violation of cyclic changes in membrane fluidity caused by a sharp increase or decrease in ambient temperature will lead to the termination of synaptic transmission, which can be recorded by the loss of the righting reflex [30]. Impaired coordination unequivocally indicates impaired nerve conduction, in particular from the optic receptor to the muscle. Indeed, disturbances in neural conduction not only in the brain, but also in peripheral neurons can cause a loss of equilibrium in the Atlantic cod *Gadus morhua* [33] Considering that each cycle of synaptic exocytosis includes reversible phase transitions of lipids of the presynaptic membrane due to entry and subsequent removal of calcium ions from the presynaptic terminal [5, 6], as well as the temperature dependence of coordination disorders [30], it can be stated that the temperature, at which loss of coordination is recorded is the temperature of the phase transition of the lipid-protein complex of the synaptic membrane. At a temperature of heat shock, the membrane remains in a state of liquid crystal, at a temperature of cold shock in a state of gel, and reversible phase transitions of lipids of a presynaptic membrane with changes in intracellular calcium are impossible. At the body level, this condition is recorded as a loss of righting reflex [30].

The conducted experiments confirmed the known linear dependence of critical temperatures (maximum and minimum) on the temperature of the long-term acclimation in the range from 20 °C to 30 °C [30, 34]. It is noteworthy that even minor deviations from definitely tolerable temperatures between 20 °C and 30 °C evoked shock reactions in fish *Danio rerio*. Shock proteins expressed both in cold and heat stress were found not only in humans but also in various species of fish, including zebrafish [35]. With a slight increase in temperature in zebrafish, the expression of hsp70 mRNA can increase tenfold, however, the expression of the HSP70 protein increases only at potentially harmful temperatures [36]. Heat shock proteins are expressed in zebrafish at 33 °C as early as 1 h after the temperature rise [36, 37]. Exposure of zebrafish to 18 °C for 4 h caused a cold stress, an increase in the content of 29 mRNA, a decrease in the content of 26 mRNA, an increase in the activity of 908 genes encoding proteins, and a decrease in the activity of 468 genes encoding proteins [38]. Note that two-thirds of the recorded range of vital activity of zebrafish *Danio rerio* (Fig. 4) lies in the region of extreme temperatures leading to the activation of stress mechanisms.

### Kinetics of temperature adaptation

Adaptation to a new ambient temperature is accomplished by regulatory reactions altering the membrane fluidity. In the frames of our notions [5, 6, 30], these processes are related with changes of intracellular Ca^2+^ concentration [14, 15] and expression of genes responsible for the processes determining the lipid composition of presynaptic membrane and synaptic vesicles [35, 37, 39]. Our experiments (Fig. 3) showed that a change in membrane fluidity occurs within a few days after being placed in an environment with a new temperature. This means that the composition of the lipid and protein components of the membrane is continuously changing for at least this time.

It was shown earlier [40–42] that new values of membrane potential and intracellular concentrations of inorganic ions were established within one hour after the switching off of the active transport. In particular, in human fibroblasts incubated in the presence of 1 µM ouabain for 1 h, membrane potential decreased from – 50 to – 10 mV [41, 42]. Calculations on the model system showed that such depolarization can cause an increase in the intracellular Ca^2+^ concentration from 10^–4^ to 10^–1^ mM, which is close to the increase of the intracellular Ca^2+^ concentration during the action potential or upon cooling by 10–15 °C [14, 15]. This means that new values of the membrane potential and ionic concentrations are established within tens of minutes.

Considering that the processes of the membrane lipid composition change may take a few days [35, 37, 39], it can be expected that the changes in T_H_ and T_C_ within the first hour after the temperature change are determined only by the current concentration of the intracellular Ca^2+^. In all plots (Fig. 3a and b), changes of T_H_ and T_**C**_ within the first hour after the sharp shift of the temperature amounted about a half of the whole range of the shock temperature changes and apparently were mostly determined by changes of the intracellular Ca^2+^ concentration [14, 15].

During adaptation to extremal high temperatures 30 °C ≤ T_A_ ≤ 38 °C, changes of the intracellular Ca^2+^ concentration did not seem to occur, but the impairment of the mechanisms responsible for the synthesis or incorporation into the membranes of the long-chain hard-melting lipids was recorded, and non-selective mechanism of the removal and utilization of lipids was functioning. The increase in T_C_ (●) and T_H_ (○) observed over several days (Fig. 3d, e) is apparently the result of an increase in the phase transition temperature of the remaining part of the membrane lipids.

If the mechanism of the lipid removal and utilization was functioning, then a decrease in T_H_(◊) to the level of 27 °C and a decrease in T_C_(♦) to 6.5 °C, which was observed within a few days (Fig. 3e and F), can be explained by a decrease in the phase transition temperature of the remaining part of the membrane lipids [5, 6]. It is of note that upper limits of the thermal niche of the fishes are very sensitive to the water hypoxia [43], however, in our experiments all aquariums with fishes were actively aerated.

It is important to note that recorded motor impairments are easily reversible. After the fish return to the vessel with a long acclimation temperature, mobility and coordination are restored within a few seconds. Note that if the characteristic times of membrane phase transitions, with regard of the establishment of the ambient temperature in the synapse region, do not exceed tens of seconds [30], then even the change in the content of intracellular ions – a process inevitably accompanying temperature changes—has characteristic times of the order of tens of minutes [40–42].

Gene expression after a change in temperature develops in the fish *Danio rerio* [37, 38] and *Carassius auratus* [44] during the first 4 h, and adaptation changes in lipid-protein complexes of synaptic membranes take tens of hours (Figs. 2 and 3). This means that the coordination disorder during a sharp change in temperature is exclusively associated with the absence of phase transitions of the membranes, which leads to the impairment of nerve conduction. An alternative assumption of impaired fusion and rupture of any other cell membranes, for example, of vascular endothelial cells, which affects many metabolic parameters, is beneath criticism, because such effects cannot be develop and disappear within a few seconds.

Long-term exposure to extreme temperatures below 10 °C or above 38 °C in our experiments was deleterious for a part of the population. Recall that in our experiments we used young fish 20–30 mm long, and such fish can tolerate extreme temperatures better than larvae or adults [45]. Fishes refused food, appeared sluggish and exhausted; abdomen was retracted, and a spinal curvature was observed. In particular, at 38 °C half of the population died out within 3 days. However, the surviving fishes could tolerate the temperature 41 °C – 42 °C for several days. A similar pattern was observed at extremely cold temperatures. It is noteworthy that the metabolism activity at 20 °C is definitely higher than at 10 °C, but fasting for several days at 20 °C did not have a significant impact on the fishes' habitus. It seems likely that the observed processes are related with disturbances in the lipid metabolism. In normal conditions, the main oxidation substrates in fishes are aminoacids, carbohydrates, and fatty acids. During adaptation to extremal temperatures in the conditions of a high O_2_ content, all ectotherms including fishes actively utilize not only lipids of the cell membranes [46] but also use lipids from fat deposits in liver and muscles [47]. It has been shown in early works that the mechanisms of interaction of lipid and protein components of cell membranes are similar in a wide variety of organisms from yeast to mammals [46, 48].

### Reversible phase transitions

The viability polygon for *Danio rerio* (Fig. 5) suggests that the temperature of the phase transition of the most hard-melting lipid-protein composition with a 1000-fold increase in [Ca^+ 2^] is reduced by (43 °C–27 °C) = 16 °C, and the temperature of the phase transition of the most low-melting lipid-protein composition with an increase in [Ca^+2^] decreases by (27–6 °C) = 21 °C. Accordingly, the temperature ranges of the phase transition are different and depend on the intracellular activity of Ca^+2^ ions. The range of temperatures of the phase transition in the lipid-protein composition at a high calcium concentration near [Ca^+2^] > 10^–1^ mM is wider than the temperature range in a calcium-free medium at [Ca^+2^] < 10^–4^ mM. The ratio of the intervals between the temperature of the thermopreferendum and the extremely high and extremely low temperatures can be estimated as 1.3.

In compliance with the notion of the reversible phase transitions of lipids of the presynaptic membrane in the process of the synaptic exocytosis [5–7, 30], it can be said that:

- The range of the phase transition temperatures of lipids of the presynaptic membrane with calcium content below 10^–4^ mM extends from 27 °C to 42 °C and amounts about 15 °C.
- The range of the phase transition temperatures of lipids of the presynaptic membrane with calcium content above 10^–1^ mM extends from 6^.^5 °C to 27 °C and is about 20 °C.

This means that a temperature decrease by 15–20 °C is equivalent to a 1000-fold increase in the intracellular calcium concentration. These results qualitatively correspond to the theoretical estimates made on the simplest model of the cell ionic-osmotic homeostasis with an account of the activity of the Na,K-APTase and Na-Ca-exchanger [14, 15]. The model predicts that at the activation energy for the ionic transport of the order of 30 kcal/mole, a cell with a membrane potential of about 60 mV at 37 °C after cooling to 26 °C, it is depolarized by 10 mV and increases the content of intracellular calcium from 10^–4^ mM to 10^–2^ mM, and a cell with membrane potential of – 60 mV at 26 °C after cooling to 7 °C, is depolarized by 20 mV and increases the content of intracellular calcium from 10^–4^ mM to 10^–1^ mM. These dependencies are analyzed in detail in works [14, 15] that provide formulas and specific graphical dependencies.

### Viability polygon

The obtained viability polygon (Fig. 5) qualitatively coincide with the results obtained for *Danio rerio* using other methods [34]. Traditionally, the polygon of the tolerance temperatures was limited by the values of the critical heat maximum Ctmax and critical cold minimum Ctmin obtained for a given fish species at different values of acclimation temperature T_A_ [34]. Determination of Ctmax implies determination of the maximal temperature, at which the fish subjected to constant linear increase in the water temperature remains alive after the return to the temperature of preliminary return to the temperature of the preliminary long-term acclimation, T_A_. Method of critical temperatures requires determination of temperatures in an immediate proximity of the temperature of the physiological death, which depends both on T_A_ and on the speed of the temperature change and even on the housing conditions of the parents [49, 50]. Maximal differences between the values of Ctmax [34] and T_**H**_ [30] are observed at T_A_ below 27 °C and between temperature Ctmin [34] and T_**C**_ [30], at T_A_ above 27 °C. The viability range determined by the methodology of loss of righting reflex [30], stretched from 6 °C to 43 °C, and the range of tolerable temperatures [34] obtained by the critical temperature method, from 4.5 to 42.0 °C. Main differences between values of Ctmax and T_H_ can be explained by incorrect determination of the temperature of physiological death. At temperatures higher than T_H_ but lower than Ctmax, the excitation propagation through the synapse is not possible [5–7, 30] and the nervous system of the organism is not able to accomplish coordination of organs and tissues, although the organs and tissues still can function independently at this temperature for some time. Similarly for temperatures lower than T_**C**_ but higher Ctmin.

In the natural area of distribution in India and Bangladesh, fishes *Danio rerio* prefer brooks, lakes, ponds, and rice fields [51, 52]. The water temperature in these reservoirs generally ranges from 16.5 °C to 34.0 °C [53]. Minimal temperature of 12.3 °C was recorded in a brook and maximal temperature of 38.6 °C, in a rice field [51, 52]. This means that the fishes are able to live at these temperatures for several hours or even days, and when possible, can leave the area of extremal temperatures and gather in the area with temperature between 20 and 30 °C.

Note that any living system emits heat and that the temperature of the fish brain in our experiments is higher than the water temperature, but not more than by 1.0 °C. However, when considering the results of other researchers, one should expect that the values of the limiting temperatures will be underestimated by several degrees. This is because brain temperature of *Danio rerio* recorded in our experiments and body surface temperature recorded in most other studies can differ by several degrees.

### Thermopreferendum temperature

In our gradient bath experiments, the thermal preferential temperature for zebrafish has been shown to be between 25 and 27 °C. This temperature of 28 °C is optimal for the cultured zebrafish embryonic cell line [54]. At this temperature, the current density in the Na^+^ channels of zebrafish myocytes and the rate of development of the action potential were similar to those in human heart myocytes at 37 °C [55]. The optimum temperature for other fish species living at a wide range of temperatures, in particular for *Lophiosilurus alexandri* [56] or *Nile tilapia* [57], is also 28 °C. Furthermore, the temperature of 25 ± 2 °C is a thermopreferendum for frogs (*Rana esculenta*) [58]. The optimal metabolic rate and the maximal rate of larva formation in nematode *Caenorhabditis elegans* [59], the maximal regeneration rate in planaria *Girardia tigrina* [60], the maximal mobility in planaria *Schmidtea mediterranea* [61] are in the same temperature range from 25 to 27 °C.

It was shown in our experiments (Fig. 3), that the temperature of the thermopreferendum T_PR_ is equal to the minimum value of the heat shock temperature (T_H_)_min_ and the maximum value of the cold shock temperature (T_C_)_max_, which corresponds to the phase transition temperature of the most hard-melting lipid-protein composition in a calcium-containing medium and the most low-melting lipid-protein composition in a calcium-free medium. It means that the revealed viability range for zebrafish 6 °C to 43 °C is employed by an overwhelming majority of living organisms found in middle latitudes.

It is difficult to explain, but interestingly the thermoneutral zone, in which the heating rate is equal to the rate of heat release into the environment, and the metabolic rate and substrate consumption are minimal, is located in the temperature range from 25.5 to 28 °C for both humans and mice [62–64], which coincides with the thermal preferendum for zebrafish.

### Arctic and tropical organisms

In arctic fish *Salvelinus alpinus*, living at 10–11 °C, the oxygen consumption and hydrogen peroxide production by mitochondria almost doubles at temperatures above 20 °C [65]. This temperature can be considered extreme for this species of fish. The ratio of the intervals between the temperature of the thermopreferendum and the highest and lowest shock temperatures equals to 1.3. According to our notion, the thermopreferendum temperature for this species is T_PR_ ≈ 11–12 °C, maximal temperature is about 20 °C, and a minimal temperature of the lipid phase transition is not lower than 5 °C. Similarly, for a cold-water trout *Salvelinus namaycush*, living at 8–10 °C, a decrease of the maximal rate of metabolism and a dramatic reduction in the metabolic recovery rate was observed at temperatures above 19 °C [66].

A tropical tilapia *Alcolapia grahami* from the lake Magadi (Kenya) lives in rapid stream sources with water temperature of up to 43 °C. The value of the critical heat maximum Ctmax, measured according to the methodology of critical temperatures [34], was 45.6 °C for this population [67]. Considering that at temperatures above 43 °C the tertiary and quaternary structure of many proteins is violated [68, 69], these results should be treated with caution. If an upper temperature limit is taken as 43 °C and thermopreferendum, as 37 °C, then a lower temperature limit, in analogy with mammalians [70, 71], will amount about 30 °C. The metabolism rate measured in these tilapias, exceeds the parameters ever recorded in ectotherms and lays within the basic range of metabolic rates of small mammals [67]. Note that the considered species live in narrow temperature ranges below 15 °C.

### Ectothermes and endothermes

The majority of mammalians and birds maintain the temperature of the visceral organs, such as brain and heart, considerably higher than the ambient temperature. To minimize the heat loss, their bodies are covered with hair or feathers. However, in different periods of life these organisms can lower the metabolic rate and, consequently, the body temperature. During the diurnal rest period, the metabolism rate estimated by oxygen consumption, in most mammalians decreases by about 20% and a body temperature, by 0.5–2.0 °C. In birds, metabolism rate can decline by 30%, and a light circadian hypothermia can reach 4.0 °C. Furthermore, a considerable number of mammalian and birds can come into a much more prolonged periods of hypometabolism and hypothermia [72]. Assuming that the thermopreferendum temperature in mammalians is 36–38 °C and the upper limit is 43 °C, then, considering that the temperature interval ratio is close to 1,0, we can expect that the lower temperature limit will be about 30 °C. This temperature is a low temperature limit recorded in many mammalian species; in particular, American black bear *Ursus americanus* during seasonal hibernation reversibly cools down to 30 °C in the circadian cycles lasting many days [70, 71]. In a closely related species, brown bear *Ursus arctos*, body temperature drops down to 6 °C and in the inter-bout arousal increases up to 30 °C [73].

Winter hibernation in many hibernators, including rodents, bats, lemurs, marsupials, hedgehogs, and brown bears (*Ursus arctos*) consists of a series of multi-day periods of catalepsy alternating with arousal periods. During catalepsy lasting 10–20 days, the body temperature is about 5–7 °C and during arousal lasting 1–2 days, the body temperature grows up to 30 °C [71, 72, 74]. In this hypometabolic state at 30 °C, in a brown bear *Ursus arctos* the metabolic activity judged by the oxygen consumption is 4 times lower than the metabolic activity in summer. The creation of similar hypometabolic state during prolonged surgical operations or under conditions of cranio-cerebral trauma in humans may turn very helpful [20].

The brain temperature of many organisms living in mid-latitudes easily varies in the range from 6 to 43 °C; the brain temperature of cold-water organisms ranges from 6 to 20 °C, and the brain temperature of most mammals and birds can vary from 30 up to 43 °C. This means that living organisms are able to exist in each of three temperature ranges, which differ in the mechanisms of synthesis, incorporation and utilization of membrane lipids. At the same time, the area of the viability polygon for cold-water species or for endothermic organisms, including humans, is 6–8 times less than the maximum possible area obtained for zebrafish in our experiments.

### Enzymatic activity

It is noteworthy that the survival range evaluated on the basis of the phase transition temperatures of lipids coincide with the functional activity ranges for most enzymes. The activities of all enzymes decrease upon cooling, and growing density of water at low temperatures, reaching maximum at 3–6 °C, favors the enzyme blockade. At these temperatures the metabolic activity virtually stops [68, 75]. At temperatures higher than 43 °C, the tertiary and quaternary structure of proteins is impaired, and this determines the upper limit of the viability range [68, 69].

The presented experimental results indicate that the upper temperature limit in cold-water fish [65, 66] and the lower temperature limit in some warm-water fish [67], mammals and birds [72–74, 76] are determined only by the phase transition temperature of membrane lipid-protein compositions, and not by a blockade of enzymatic activity.

### Exclusive integral parameter

At the cellular and brain levels, there are numerous complex signaling mechanisms. However, a violation in the processes of coordination of the activity of different muscles of the fish body, recorded in our behavioral experiments, unambiguously indicates that signals in the form of sequences of nerve impulses from the visual receptor or from the vestibular apparatus do not enter at least one muscle of the body. The impairment of coordination will be recorded for any disorder in nerve conduction, both at the receptor level, and in various brain structures or at the level of the neuromuscular synapse.

It is important to note that recorded motor impairments are easily reversible. After the fish return to the vessel with a long acclimation temperature, mobility and coordination are restored within a few seconds. Note that if the characteristic times of membrane phase transitions, with regard the time of the establishment of the ambient temperature in the synapse region, do not exceed tens of seconds [30], then even the change in the content of intracellular ions has characteristic times of the order of tens of minutes [40–42]. Gene expression after a change in temperature develops in the fish *Danio rerio* [37, 38] or *Carassius auratus* [44] during the first 4 h, and adaptation changes in lipid-protein complexes of synaptic membranes take tens of hours (see Fig. 3). This means that the coordination disorder during a sharp change in temperature is exclusively associated with the absence of phase transitions of the membranes, which leads to the impairment of nerve conduction. An alternative assumption of impaired fusion and rupture of any other cell membranes, for example, of vascular endothelial cells, which affects many metabolic parameters, is beneath criticism, because such effects cannot be develop and disappear within a few seconds.

Our experiments (see Figs. 1 and 4) showed that any investigated T_A_ corresponded to well-defined temperatures of phase transitions T_C_ and T_H_. Many researchers have long tried to determine the effect of the temperature of long-term acclimation T_A_ on the components of lipid membrane [77–85]. It has been shown that adaptation to any T_A_ occurs simultaneously with changes in many combinations of lipid and protein components. A comparative analysis of the fatty acid composition of the brain of 17 species of teleost fish obtained from Antarctic, temperate and semi-tropical waters, as well as from rats, turkeys, mammals and birds as typical was carried out. Analysis of the lipid composition of the brain has shown that the relative amounts and ratios of different lipids are the main factors contributing to the maintenance of proper fluidity [77, 78]. Cold adaptation correlated with an increase in the proportion of unsaturated fatty acids, mainly polyunsaturated fatty acids [77], as well as highly unsaturated phospholipids [79]. The complexity of the analysis of the mechanisms of membrane homeostasis is determined by the presence of many mechanisms that control the properties of the membrane and adjust its composition [80]. Note that some lipids are intermediate products of cellular metabolism and modulators of certain metabolic pathways [81]. Moreover, not only proteins, but also peptides, change the structure of the membrane, creating domains of liquid crystals in the membrane [82], and the activity of membrane enzymes, in turn, depend on the fluidity and lipid composition of the membranes [80]. A change in temperature of the membrane phase transition can occur, for example, when a part of cholesterol is transferred from the plasma membrane to the endoplasmic reticulum [83] or when cholesterol-independent domains are formed under the action of saturated long-chain phospholipids C24 and C16 [84]. It is assumed that temperature adaptation of the brain is associated with the aggregation of monomers of acetylcholinesterase and its dissociation of their tetramers [85]. The task is complicated by the fact that more than 40.000 lipids can be identified [86] and more than 5.000 different membrane proteins [87] were detected in the composition of cell membranes. It is practically impossible to predict the behavior of such a complex system from the standpoint of biochemistry.

The listed facts allow us to state that many variants of lipid and protein components of synaptic membranes can correspond to any specific T_A_ value. Moreover, our experiments (Fig. 3) showed that the change in membrane fluidity occurs within a few days after being placed in an environment with a new temperature. This means that the composition of the lipid and protein components of the membrane is continuously changing for at least this time. Many variants of the composition of lipid and protein components of the synaptic membrane seem to depend on the type of organism and on the temperature prehistory (heating or cooling) of the nervous system.

The loss of righting reflex methodology allows us to characterize the state of the membrane using only one parameter. The advantage of our method of recording temperature shock is the possibility of detecting phase transitions of membrane lipids in a behavioral experiment in vivo. We assume that the minimum phase transition temperature of the most low-melting lipid-protein composition at a high calcium concentration does not exceed 7 °C, and the maximum phase transition temperature of the highest-melting lipid-protein composition is 27 °C, which is the minimum phase transition temperature of the most low-melting lipid protein composition in a calcium-free medium. The maximum temperature of the phase transition of the most refractory lipid-protein composition in a calcium-free medium is 43 °C, and at a high calcium concentration it is 27 °C. Mammals, birds, and many organisms from polar or tropical waters live in much narrower temperature ranges.

Apparently, narrow temperature ranges of vital activity correspond to narrower temperature ranges of phase transitions and different values of the thermopreferendum. The phase transition temperature is the exclusive integral parameter characterizing the functional state of membrane lipids.

## Conclusions

The use of a fish *Danio rerio* and the loss of righting reflex methodology made it possible to determine the limits of temperature adaptation in terms of temperatures of cold shock and heat shock.

It is shown that the experimental values of the temperatures of cold shock and heat shock and the temperature of the thermal preferendum correspond to the temperatures of phase transitions of the lipid-protein composition of the synaptic membrane between the liquid and solid states. The resulting range of viability coincides with the temperature range in which cellular enzymes function effectively.

**Fig. 6:**
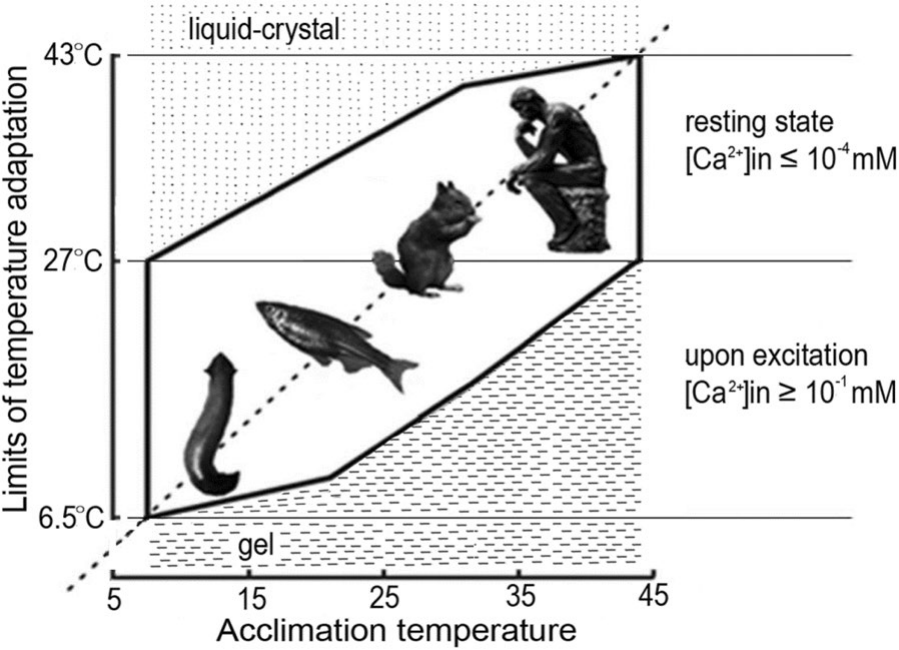
The limits of temperature adaptation of various organisms. *Abscissa*, acclimation temperature T_A_[^o^C], and *ordinate,* characteristic constants: (T_C_)_min_[^o^C], T_PR_[^o^C] and (T_A_)_max_[^o^C]

The results of our experiments allowed us to give a unified interpretation of fragmentary and disparate experimental results of other researchers. The analysis revealed important characteristic constants that determine the polygon of viability of many organisms: (T_C_)_min_ = 7 °C, (T_H_)_max_ = 43 °C, T_PR_ = (T_C_)_max_ = (T_H_)_min_ = 27 °C, as well as the ratio of the intervals between the thermal preferendum and extremely low and extremely high temperatures, which is close to 1.3. The viability polygon and the ratio of cold shock to heat shock temperature ranges obtained for *Danio rerio* describe with good accuracy those for the planarian *Girardia tigrina*, the ground squirrel *Sermophilus undulatus*, and for *Homo sapiens* (Fig. 6).

At the genome level, the overwhelming majority of organisms are able to create lipid-protein compositions of synaptic membranes, the melting temperature of which can vary from 7 to 27 °C in presence of 10^–1^ mM calcium ions and from 27 to 43 °C, in a calcium-free environment. In fish and planarians, this ability is preserved throughout life, while in mammals, including humans, only in early postnatal ontogenesis, and in hibernators this occurs periodically, during seasonal hibernation.

The polygon of the viability of a particular organism is determined not only by the genome, but also by the physicochemical properties of lipids that make up the membrane structures of synaptic endings. The limits of temperature adaptation of any biological species are determined by the temperature range of the functioning of its nervous system.

## Data Availability

All data and materials are available.

## Abbreviations

T_A_: Acclimation temperature
T_H_: Heat shock temperature;
T_C_: Cold shock temperature
(T_C_)_min_: Minimum temperature of cold shock
(T_C_)_max_: Maximum temperature of cold shock
(T_H_)_min_: Minimum temperature of heat shock
(T_H_)_max_: Maximum temperature of heat shock
T_TP_: Thermopreferendum temperature
